# RETRACTED: Naamneh et al. Structure–Activity Relationship of Synthetic Linear KTS-Peptides Containing Meta-Aminobenzoic Acid as Antagonists of α1β1 Integrin with Anti-Angiogenic and Melanoma Anti-Tumor Activities. *Pharmaceuticals* 2024, *17*, 549

**DOI:** 10.3390/ph18091400

**Published:** 2025-09-18

**Authors:** Majdi Saleem Naamneh, Tatjana Momic, Michal Klazas, Julius Grosche, Johannes A. Eble, Cezary Marcinkiewicz, Netaly Khazanov, Hanoch Senderowitz, Amnon Hoffman, Chaim Gilon, Jehoshua Katzhendler, Philip Lazarovici

**Affiliations:** 1 School of Pharmacy Institute for Drug Research, Faculty of Medicine, The Hebrew University of Jerusalem, Jerusalem 9112002, Israel; majdi.naamneh@mail.huji.ac.il (M.S.N.); michal.klazas@mail.huji.ac.il (M.K.); amnonh@ekmd.huji.ac.il (A.H.);; 2 VINČA Institute of Nuclear Sciences, National Institute of the Republic of Serbia, University of Belgrade, Mike Petrovića Alasa 12–14, 11000 Belgrade, Serbia; momict@gmail.com; 3 Institute of Physiological Chemistry and Pathobiochemistry, University of Münster, Waldeyer-Str. 15, 48149 Münster, Germanyjohannes.eble@uni-muenster.de (J.A.E.); 4 Debina Diagnostics Inc., 33 Bishop Hollow Rd., Newtown Square, PA 19073-3211, USA; cmarcink@temple.edu; 5 Department of Chemistry, Bar Ilan University, Ramat-Gan 5290002, Israel; netalyk@gmail.com (N.K.); hsenderowitz@gmail.com (H.S.); 6 Institute of Chemistry, The Hebrew University of Jerusalem, Jerusalem 9190401, Israel; chaimgilon@gmail.com

The journal retracts the article titled “Structure–Activity Relationship of Synthetic Linear KTS-Peptides Containing Meta-Aminobenzoic Acid as Antagonists of α1β1 Integrin with Anti-Angiogenic and Melanoma Anti-Tumor Activities” [1], cited above.

Following publication, concerns were brought to the attention of the Editorial Office regarding a range of image irregularities contained within this article [1].

Adhering to our standard procedure, an investigation was conducted by the Editorial Office and Editorial Board that identified indications of inappropriate editing and partial duplication between figures presented in this article [1] and earlier publications [2,3], produced by a different research group. While the authors collaborated within this process, raw material meeting the journal’s requirements for original images could not be provided for Editorial Board evaluation (https://www.mdpi.com/journal/molecules/instructions#oriimages, accessed on 20 August 2025). Consequently, the Editorial Board has lost confidence in the reliability of the findings and has decided to retract this publication, as per MDPI’s retraction policy (https://www.mdpi.com/ethics#_bookmark30, accessed on 20 August 2025).

This retraction was approved by the Editor-in-Chief of the *Pharmaceuticals* journal.

Johannes A. Eble agrees to this retraction. The remaining authors disagree with this retraction.
